# Safety and Pharmacogenetics of Oxycodone in Post-Cesarean Analgesia and Breastfeeding Dyads: A Proactive Approach to Precision Medicine

**DOI:** 10.3390/healthcare14010093

**Published:** 2025-12-31

**Authors:** Snehi Shetal Shah, Hsing-Hua Sylvia Lin, Sauren Baheti, Erin Bundock, Alex Anderson, Rose Barlow, Barkha Patel, Linda Park, Senthilkumar Sadhasivam

**Affiliations:** 1 University of Toledo College of Medicine and Life Sciences, Toledo, OH 43614, USA; snehi.shah@rockerts.utoledo.edu; 2 Department of Anesthesiology and Perioperative Medicine, University of Pittsburgh, Pittsburgh, PA 15224, USA; hsl26@pitt.edu (H.-H.S.L.); bahetis@upmc.edu (S.B.); bundocke@upmc.edu (E.B.); andersonah2@upmc.edu (A.A.); barlowr@upmc.edu (R.B.); patelb9@upmc.edu (B.P.); yup17@pitt.edu (L.P.); 3 Clinical and Translational Science Institute, University of Pittsburgh, Pittsburgh, PA 15224, USA

**Keywords:** precision medicine, opioid-sparing, oxycodone, pharmacogenetics, CYP2D6, cesarean delivery, postoperative nausea and vomiting, pain, neonatal outcomes

## Abstract

**Background**: The aim of the study is (1) to assess safety of opioids in nursing mothers after cesarean delivery and in breastfed infants and (2) to evaluate the role of CYP2D6 genetics in maternal and infant clinical outcomes after cesarean delivery. **Methods**: A total of 210 mother–infant dyads were enrolled after cesarean delivery. Oxycodone 5 mg orally was administered every 4–6 h as needed as part of a standardized opioid-sparing ERAS protocol. Primary outcomes were opioid-related adverse effects, including maternal respiratory depression (RD) and postoperative nausea and vomiting (PONV) and neonatal composite side effects (i.e., RD monitoring, sedation, and limpness). **Results**: In total, 77% of mothers received opioids during postpartum hospital stay, none experienced respiratory depression, 13% reported PONV, and composite opioid-related side effects were observed in 13% of neonates. Compared to mothers without opioid consumption, higher in-hospital opioid consumption was borderline significantly associated with a higher risk of neonatal composite side effects (adjusted relative risk, aRR = 3.79; 95%CI: 1.01–14.28; *p* = 0.07), with a similar trend toward higher risk in maternal PONV (aRR = 2.56; 95%CI: 0.70–9.29; *p* = 0.36). Mothers with a CYP2D6 ultra-rapid metabolizer phenotype also showed higher rates of PONV and neonatal composite side effects compared with normal or intermediate phenotypes, although these associations were not statistically significant. **Conclusions**: Higher maternal in-hospital opioid consumption is associated with a higher risk of neonatal composite side effects. Using the lowest effective doses of opioids as needed could reduce the risk of opioid-related side effects in neonates. Preoperative genotyping may help identify mothers and breastfed neonates at increased risk for opioid-related adverse outcomes. Additional studies are needed to evaluate preoperative genotyping and to evaluate the causality of increased neonatal adverse outcomes.

## 1. Introduction

Cesarean delivery (CD) is one of the most common surgical procedures worldwide, comprising 32% of births in the United States and over 1.2 million procedures annually [1]. The rising prevalence of CD is attributed to advances in surgical techniques, preferences for elective repeat CD, and an increase in high-risk pregnancies linked to obesity, diabetes, hypertension, and advanced maternal age [2,3]. Surveys indicate that the primary concern surrounding CD for patients is both intraoperative and postoperative pain, followed by nausea and vomiting [4]. These factors emphasize the growing importance of comprehensive postoperative pain management in modern obstetric care.

Adequate postoperative pain management is imperative not only for maternal recovery, but also for breastfeeding success and neonatal bonding [5,6]. Inadequate pain management may delay maternal recovery, limit mobility, affect psychological well-being, and impair mother–infant bonding, which is vital for neonatal development [5,7].

Current management guidelines recommend a multimodal analgesia approach integrating several methods to optimize pain relief and mitigate side effects. Pain control methods include long-acting neuraxial opioid analgesics, non-opioid analgesics such as acetaminophen and nonsteroidal anti-inflammatory drugs (NSAIDs), transversus abdominis plane blocks (TAP), and postoperative oral opioid prescriptions [8]. Neuraxial anesthesia, often paired with preservative-free morphine, is the preferred intraoperative method due to its extended postoperative analgesia, minimized maternal–fetal risks, and avoidance of airway complications associated with general anesthesia. Patients typically receive neuraxial anesthesia with local anesthetics administered intrathecally or epidurally, which are commonly combined with a long-acting opioid such as preservative-free morphine to provide analgesia over the first 24–48 h postoperatively. At the end of surgery, TAP blocks are often administered to provide augmented localized abdominal pain relief.

Postoperatively, pain management is then tailored to an individual patient’s pain experience and is centered around a combination of oral opioids, NSAIDs, and acetaminophen [9]. Despite efforts to reduce opioid reliance, over 80% of postpartum women still require opioids, with oxycodone being a common choice due to its potency and efficacy in addressing acute postoperative pain [10]. However, oxycodone poses unique risks to breastfeeding neonates, including sedation, respiratory depression (RD), and potential developmental effects [11,12].

Despite these concerns, evidence-based guidelines for the safe use of opioids like oxycodone in this population remain limited. Additionally, current postoperative pain management guidelines, while effective, are not without significant risk, and do not account for variations in genetic polymorphisms that play a role in opioid metabolism [7]. The metabolism of opioids is influenced by genetic polymorphisms, particularly in the CYP2D6 enzyme [9]. CYP2D6 variations classify individuals as poor, normal, or ultra-rapid metabolizers (UMS), significantly affecting drug metabolism [11,13]. UMs convert opioids like codeine and tramadol into active metabolites more rapidly, increasing the risk of neonatal sedation or RD through breast milk exposure [11]. The impact of these metabolism variations were exemplified in a case involving a healthy full-term neonate that experienced lethargy and difficulties in breastfeeding which progressed to death due to maternal UM status ultimately resulting in unsafe quantities of morphine in the breast milk [12].

In response to the 2017 FDA warnings regarding the use of codeine and tramadol in breastfeeding mothers, clinical practices have shifted to prescribing alternative opioids such as oxycodone and hydrocodone instead of codeine and tramadol for nursing mothers [14,15]. Although these opioids are structurally different, they are also metabolized by CYP2D6 and, therefore, are equally influenced by CYP2D6 genetic polymorphisms potentially heightening risks in UMs. In fact, the CYP2D6 metabolites of oxycodone (oxymorphone) and hydrocodone (hydromorphone) are 5–14 fold stronger than the CYP2D6 metabolites of codeine and tramadol [15]. Given oxycodone’s potency and metabolism by the CYP2D6 pathway, UMs who potentially metabolize opioids more quickly and produce increased levels of active metabolites in circulation may promote elevated risks of adverse effects such as sedation or RD in neonates [16]. Conversely, poor metabolizers may require higher doses to achieve effective pain control, increasing the susceptibility to prolonged opioid use or misuse. This shift in clinical practice to stronger opioids could place new mothers and their breastfed infants at greater risk of adverse effects.

This study aims to evaluate the safety and efficacy of oxycodone for post-CD pain management in breastfeeding mothers and their neonates and to investigate the predictive role of pharmacogenetic markers, particularly CYP2D6, in maternal and neonatal clinical outcomes. Findings from this work will support the development of personalized analgesic regimens to enhance safety and efficacy while addressing the unique challenges of this vulnerable population.

## 2. Materials and Methods

### 2.1. Study Design and Population

This prospective, genotype-blinded observational study was conducted at a tertiary care center to enroll at least 200 mother–infant dyads. Participants were recruited following scheduled and emergent CDs and were required to meet specific inclusion criteria, including the plan to breastfeed and the ability to provide informed consent. Mothers with contraindications to opioid use or known hypersensitivity to study drugs were excluded. Ethical approval was obtained from the Institutional Review Board at the University of Pittsburgh (STUDY21070164), and all participants consented to genetic testing as part of the study.

### 2.2. Pain Management Protocol

Postoperative pain management followed a standardized Enhanced Recovery After Surgery (ERAS) protocol, emphasizing multimodal analgesia to reduce opioid reliance [17]. Mothers received oxycodone 5 mg every 4–6 h as needed for pain, supplemented by non-opioid analgesics such as acetaminophen and NSAIDs. Neonatal monitoring included standardized assessments for signs of sedation, RD, and other opioid-related adverse effects.

### 2.3. Outcome Measures

Maternal outcomes included the incidence of respiratory depression, postoperative nausea and vomiting (PONV), and pain assessed by the Numerical Rating Scale (NRS). RD was defined as a persistent (>1 min) oxygen desaturation (SpO2) < 90% on room air or respiratory rate < 8 breaths per minute or SpO2 < 94% along with a respiratory rate of <10 per minute requiring supplemental oxygen to maintain SpO2 > 94% in the absence of upper airway obstruction. Respiratory rate and oxygen saturation were collected from medical records and clinical vitals during pre-op, pacu, and inpatient stay. Assessment for PONV was scored as a binary variable (yes or no) during in person patient assessments. Nausea was defined as the feeling of wanting to vomit. Vomiting was defined as the forceful expulsion of stomach contents. These assessments were collected during pre-op pacu and daily during inpatient stay.

Additional metrics included total in-hospital opioid consumption, measured in morphine milligram equivalents (MMEs), and length of hospital stay. Neonatal outcomes include respiratory depression, sedation, and limpness. Maternal reporting of neonatal sedation, defined as prolonged sleep (>4 h), was assessed for mothers that consented to this data collection. Respiratory rate was taken every 8 h from EMR. Composite adverse effect was categorized as “Yes” if a neonate experienced at least one of three opioid-related side effects (respiratory depression, sedation, and limpness) from postpartum day 0 to day 3.

### 2.4. Genetic Analysis

Blood draws for genotyping: The first genotyping maternal blood sample was collected at a regularly scheduled prenatal visit at gestational weeks 27–37 or was obtained preoperatively during the day of surgery. This blood collection consisted of a 1 EDTA tube (lavender top) of approximately 3 mL of blood. The infant blood sample (lavender top) consisted of approximately 1–3 mL of cord blood and was collected from the cord during surgery. Maternal and neonatal DNA samples were analyzed for CYP2D6 genetic polymorphisms using the PharmacoScan Solution assay (ThermoFisher, Waltham, MA, USA). Applied Biosystems™ Axiom™ Analysis Suite software (Version 5.4) was used to translate CYP2D6 genotypes to metabolizer phenotypes, which included normal, intermediate, poor, ultra-rapid, or unknown metabolizers.

### 2.5. Statistical Analysis

Sample size and power justification: We planned to recruit 200 breastfeeding newborn-mother dyads. The type I error was fixed at 0.05 and we specified a minor allele frequency of 0.2, infant’s oxycodone related adverse effects of 20%, mother’s adverse drug event rate of 60%, and a relative risk of 2. The 200 samples will yield a power >80% for detecting infant and maternal opioid-related adverse effects using additive models.

Continuous variables were summarized as means with standard deviations (SDs) or as medians with 25th and 75th percentiles, as appropriate. Categorical variables were summarized as counts and percentages. Group comparisons were conducted using the Kruskal–Wallis test for continuous variables and the Chi-square test for categorical variables. Generalized estimating equation (GEE) models with a log link and binomial distribution were used to estimate relative risks (RRs) and 95% confidence intervals (CIs) for binary primary outcomes (i.e., PONV and neonatal composite outcome of opioid side effects). GEE models with a log link and gamma distribution were used to estimate mean ratios and 95% CIs for continuous total opioid MME outcomes. Adjusted models included total opioid MME, maternal CYP2D6 metabolizer phenotype, average pain score over three postpartum days, and prespecified maternal confounders (age, race, BMI, and any mental illness). Statistical significance was defined as a *p* < 0.05. All analyses were performed using SAS 9.4 (SAS Institute, Cary, NC, USA).

## 3. Results

### 3.1. Baseline Characteristics

A total of 210 mothers were enrolled in this prospective cohort study, with a mean age of 32 years. The cohort was 80% White, 12% Black, and 5% Hispanic (Table 1). The mean BMI was 34.6 kg/m^2^, with a median gravity of 2 and median parity of 1. Most mothers (83%) underwent scheduled cesarean delivery, of whom 85% received spinal anesthesia and 11% received anesthesia via a pre-existing epidural. The mean surgical duration was 52 min. The prevalence rate of any mental illness was 56%, most commonly anxiety or depression (55%), followed by PTSD (7%) and ADHD (4%) (Table 1).

### 3.2. CYP2D6 Metabolizer Phenotypes

Maternal CYP2D6 phenotypes indicated that 91% were normal or intermediate metabolizers, 6% were poor metabolizers, and 4% were ultra-rapid metabolizers (Table 1). Among neonates, 4% were poor metabolizers and 2.6% were ultra-rapid metabolizers (Appendix A Table A1). Maternal and infant CYP2D6 phenotypes were not highly concordant among the 190 infant-mother pairs with available phenotype results (Appendix A Table A1).

### 3.3. Maternal and Neonatal Clinical Outcomes

Table 2 summarizes any maternal and neonatal clinical outcomes experienced across PPD0-3. Among the 210 enrolled mothers, none experienced respiratory depression and 13% reported at least one episode of PONV, which occurred primarily during PACU on PDD 0 (Appendix A). The average NRS pain score was 3.2 (SD = 1.6) over PPD0-3, with a mean max pain score of 4.9 (SD = 1.9) (Table 2). Pain scores peaks on postoperative day 2 (mean NRS 4.0 ± 2.0) and declined to 3.3 ± 1.8 by PPD3 (Appendix A). The mean total opioid consumption in the hospital was 44.4 (SD = 47.5) MME (mg) among 210 mothers (Table 2). A total of 161 mothers (77%) received any in-hospital opioids, with an average daily opioid consumption of 21.9 (SD = 19.3) MME (mg) on PPD 2 (Appendix A Table A2). More than 60% of mothers received opioids in the first 48 h postpartum, and it decreased to 29% by PPD 3 (Appendix A Table A2).

Among neonates, 28 (13%) experienced at least one opioid-related adverse outcome, including monitoring of respiratory depression (n = 8), sedation (n = 7), and limpness (n = 15) over PPD 0–3. In addition, 37% (n = 77) neonates had extended length of stay > 72 h and three neonates (1%) exhibited opioid withdrawal symptoms, defined as SOWSS > 4 during hospital stays (Table 2).

### 3.4. Relationship Between Opioid Consumptions and Maternal CYP2D6 on Clinical Outcomes

**Figure A1:**
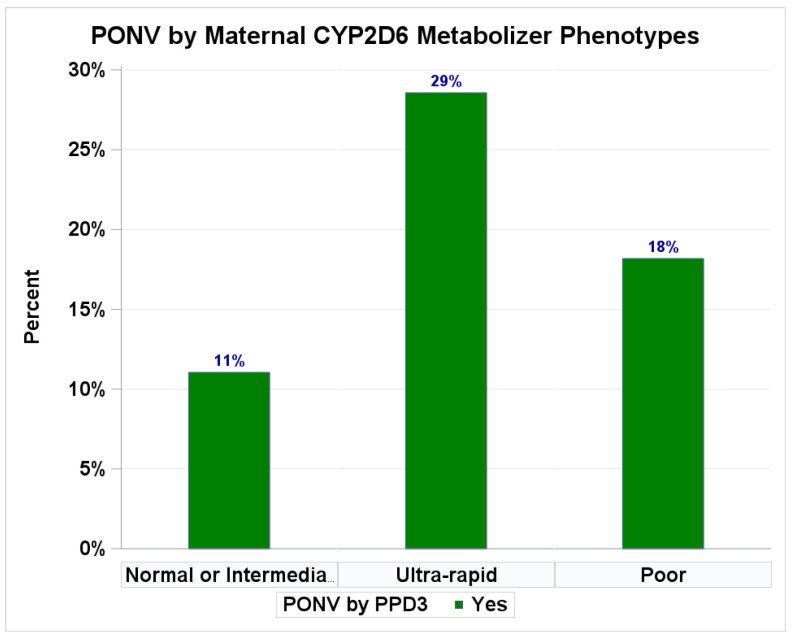
Clinical outcomes by maternal CYP2D6 metabolizer phenotypes. Percentages of PONV.

Higher rates of maternal PONV were observed among those with greater in-hospital opioid consumption (>70 MME: 16.3% PONV) compared to those with no opioid use (8.2% PONV) (Table 3) and among those with a CYP2D6 ultra-rapid phenotype (28.6% PONV) compared to normal or intermediate metabolizers (11.0% PONV) (Table 3 and Appendix A Figure A1). Higher opioid consumption (>70 MME) showed a trend toward an increased risk of maternal PONV (adjusted RR = 2.56; 95%CI: 0.70–9.29; *p* = 0.36), after accounting for total opioid consumptions, CYP2D6 phenotype, average pain score over three postpartum days, age, race, BMI, and any mental illness.

**Figure A2:**
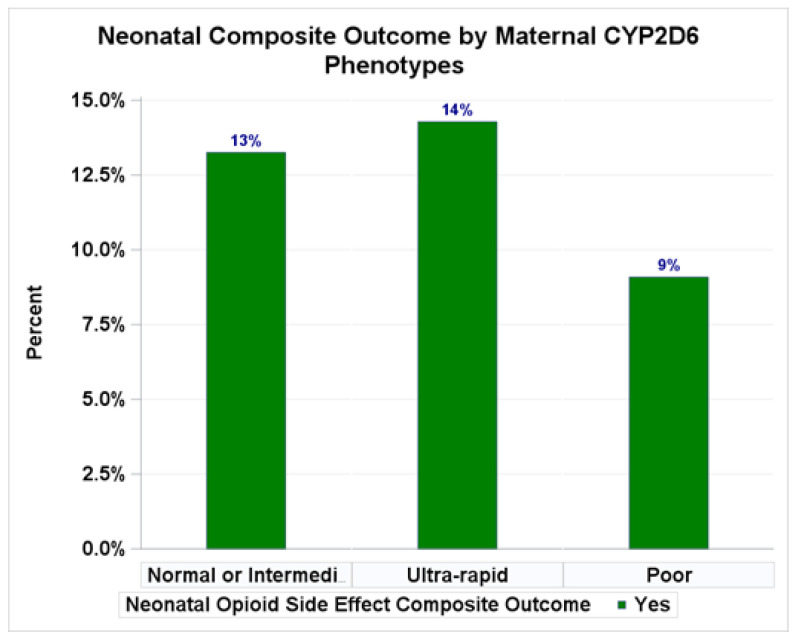
Clinical outcomes by maternal CYP2D6 metabolizer phenotypes. Percentages of neonatal composite outcomes.

We also observed higher rates of neonatal composite outcomes from opioid side effects (RD monitoring, sedation, and limpness) among those with greater in-hospital opioid consumption (>70 MME: 22.4% opioid side effects) compared to those with no opioid use (6.1% opioid side effects) (Table 3) and among those with a CYP2D6 ultra-rapid phenotype (14.3% opioid side effects) compared to poor metabolizers (9.1% opioid side effects) (Table 3 and Appendix A Figure A2) without being statistically significant in the unadjusted models. Compared to those with lower or no opioid consumption, the adjusted model indicates a boarderline significant relationship between higher in-hospital opioid use and neonatal composite outcomes from opioid side effects (>70 MME: adjusted RR = 3.79, 95%CI: 1.01–14.28, *p* = 0.07)

**Figure A3:**
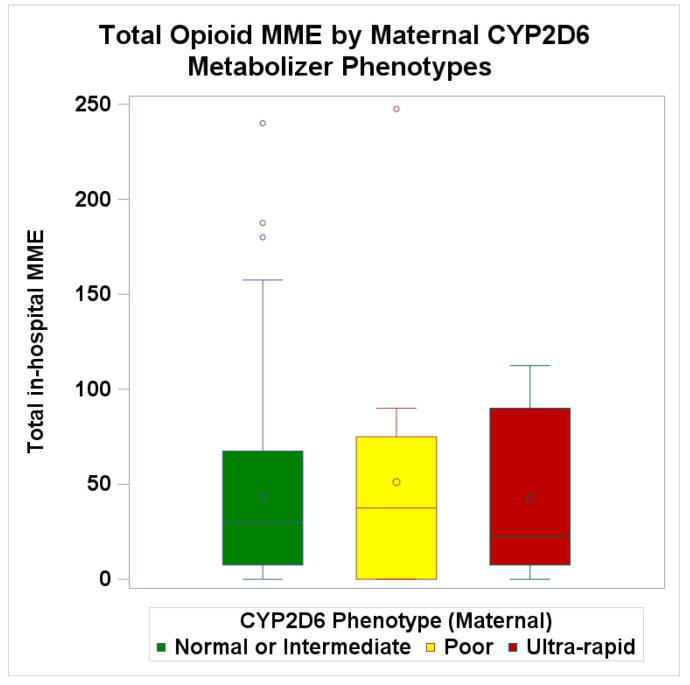
Clinical outcomes by maternal CYP2D6 metabolizer phenotypes. Boxplot of average total opioid use over postpartum three days.

Mothers who reported an average pain score of 4 and above over the first three postpartum days required higher opoid use (78.6 MME in NRS 4+ vs. 31.3 MME in 0–3), and this association remained statistically significant in the adjusted model (adjusted mean ratio: 2.44; 95%CI: 1.29–4.61; *p* = 0.01) (Table 4). Opioid consumption was similar by CYP2D6 status (Table 4 and Appendix A Figure A3). Poor metabolizers required slightly higher total opioid consumption (mean MME: 51.1) compared to normal or intermediate (mean MME: 43.2) and ultra-rapid metabolizers (mean MME: 42.9). The adjusted mean ratio was 1.09 (95%CI: 0.34 to 3.50; *p* = 0.92) for poor metabolizers compared to normal or intermediate metabolizers (Table 4).

## 4. Discussion

In this prospective study of 210 nursing mother–neonate dyads, 77% of mothers received opioids during their postpartum hospital stay. No mothers experienced respiratory depression, although 13% had at least one episode of PONV. Neonatal composite opioid-related side effects were documented in 13% of neonates. Relative to mothers who did not receive opioids, those with higher in-hospital opioid consumption appeared more likely to have neonates with recorded composite events. Higher maternal opioid use had a trend, although non-significant, toward increased maternal PONV. Given the observational design, the strong correlation between pain severity and opioid use, and the neonatal composite outcomes not specific to opioids, these patterns should be interpreted as hypothesis-generating signals rather than evidence of a causal opioid effect.

Exploratory analyses of the CYP2D6 phenotype suggested that mothers classified as ultra-rapid metabolizers may have higher rates of PONV and neonatal composite side effects compared with other phenotypes; however, these comparisons were based on very small numbers of ultra-rapid and poor metabolizers and were not statistically significant, and therefore warrant confirmation in larger, adequately powered cohorts.

### 4.1. Maternal Safety and Efficacy

The absence of respiratory depression in mothers receiving oxycodone within the ERAS protocol supports its safety when used in carefully monitored settings. However, variability in pain scores and opioid requirements across the CYP2D6 phenotype may suggest the importance of personalized analgesic strategies. Poor metabolizers required higher opioid doses, raising concerns about the potential for prolonged use and adverse events in this subgroup [18,19]. Conversely, UMs reported higher peak pain scores despite requiring lower doses, suggesting that standard dosing regimens may not adequately address breakthrough pain in this group.

### 4.2. Neonatal Safety

The findings raise important concerns about the safety of maternal oxycodone use during breastfeeding. The high rates of composite neonatal opioid-side effects (RD, limpness, and sedation), particularly in infants of mothers with ultra-rapid metabolizers status, warrant careful clinical consideration. The hypothesis-generating association between maternal CYP2D6 status and neonatal outcomes highlights the potential utility of preoperative genotyping in identifying at-risk dyads. Such measures could enable the proactive tailoring of analgesic regimens to minimize neonatal exposure to opioids upon greater analysis of a larger sample size.

This study focuses on the incidence of adverse events as a function of the CYP2D6 phenotype as a proxy for CYP2D6 enzymatic activity. Other studies have observed changes in neonatal CYP2D6 concentrations among infants of varying sex, ethnicity, gestational ages, and genotypes, and found that increasing gestational age is associated with CYP2D6 activity and protein content [20]. The hepatic expression of many CYP450 enzymes is typically between 50 and 70% of adult values at term gestation, with enzymatic activity increasing with postnatal age [21]. CYP2D6 increases steadily throughout the first week of life; however, by 5 years of age, only reaches approximately two-thirds of adult values [21].

Decreased plasma protein binding, differences in volume of distribution, and the immaturity of hepatic metabolic enzymes all contribute to a lower margin of safety and an increased risk of adverse effects of opioids in neonates than adults. The significant individual variability of expression of CYP enzymes within neonates further complicates the establishment of safe dosing [22]. Individual and small dose titrations may be the best practice due to the large interindividual variability, despite the half-life and clearance generally increasing with postnatal age [23]. The elimination half-life is longest and clearance is slowest in the newborn period (0–7 days of life) as compared to older infants [24]. Despite these concerns, oral oxycodone metabolites in breastmilk are generally well-tolerated by neonates. This could be partially due to poor enteric absorption in the neonatal period [21]. However, more studies are needed in order to establish the safe dosing curves of oral opioids in this population.

### 4.3. Study Limitations

This study was conducted at a single center with a relatively small sample size, which limits both the power and generalizability of our findings. Due to the methods of measuring our primary neonatal outcomes, there may be variability in the incidence, scoring, and timeframe of adverse effects seen. Overall, there were few participants who were lost to follow up in this study during the hospital stays. However, future studies should consider the long-term follow up of neonates both in first year of life and further developmental outcomes. This study did not account for maternal factors such as opioid-naive or opioid-sensitive patients. Patients who are opioid-dependent or have previous exposure to opioids may require increased postoperative oxycodone doses for pain management and therefore may have increased metabolites in their breast milk. Cultural attitudes toward pain management can also influence opioid use, potentially introducing bias or variability in outcomes. Large variability has been seen in morphine pharmacokinetics in terms and preterm infants; however, the clearance of morphine rate has been estimated to increase by approximately 0.9 mL/kg/min per week of gestation. Therefore, it is likely that a higher frequency of adverse events may be seen in premature infants at exposure to similar levels of oxycodone metabolites in breastmilk [25].

In interpreting the observed association between higher maternal opioid consumption and the neonatal composite outcome, confounding by maternal pain severity is likely a contributing factor. Mothers with more severe pain appropriately receive higher opioid doses as part of routine clinical care, so opioid dose is essentially a marker of underlying pain burden rather than an independent exposure assigned at random. Higher opioid requirement may also be indicative of greater maternal distress, breastfeeding difficulties, and maternal mental illnesses such as anxiety and depression. As a result, neonates of mothers with higher pain scores are more likely to be observed for possible sedation, limpness, or respiratory depression, even when the underlying physiology may not differ meaningfully from other infants despite standard clinical care. This pattern introduces both confounding by indication and surveillance bias, and therefore the association between maternal opioid dose and neonatal adverse events should not be interpreted as evidence of a direct causation. Although our multivariable models adjusted for pain severity, mental health illness, and several maternal and obstetric characteristics, residual confounding from unmeasured aspects of breastfeeding dynamics, and severe anxiety or depression is likely to persist.

An additional limitation of this study is the absence of detailed data on breastfeeding patterns and quantification of milk production and feeding. Neonatal opioid exposure via breast milk not only depends on the maternal dose and pharmacogenetic profile, but also on the feeding frequency, the timing of feeds relative to maternal dosing, and the volume and composition of the breast milk produced and ingested. We did not collect standardized information on lactation onset, the degree of breastfeeding versus formula supplementation, the timing of feeds around oxycodone administration, or the maternal factors that may impair milk production. Without these data and maternal blood and breast milk concentrations of opioids, we cannot accurately estimate individual neonatal opioid exposure or examine dose–response relationships of opioid exposure via breast milk intake and neonatal adverse events.

This study was unable to account for all of the genetic polymorphisms that have been identified as being involved in the pharmacokinetics of oxycodone, as well as those that play a role in pain sensitivity, the transport of opioids across the blood–brain barrier, stress or inflammatory markers, or the epigenetic effects of pain and analgesia, especially those involved in pregnancy. For example, the enzyme CYP3A7 is highly expressed in fetal hepatocytes, with levels remaining high in the first week of life. This could be protective against adverse effects of opioids, as it has a higher affinity and maximal velocity than CYP3A4, which is present in adult liver tissues [26]. Furthermore, there are some polymorphisms that show an indirect relationship between opioid-induced side effects and analgesic effects which may imply a more complex genetic process at work [27]. These pharmacogenetics and combinatorial genetics may be interesting points of further studies. In addition, we did not obtain pharmacokinetic measurements of oxycodone or its active metabolite, oxymorphone, in maternal plasma or breast milk, nor did we assay neonatal drug levels. The absence of these exposure data limits our ability to directly link maternal genotype and dose to actual metabolite concentrations and neonatal risk, and therefore our pharmacogenetic findings should be interpreted as hypothesis-generating associations rather than causal evidence.

### 4.4. Study Strengths and Clinical Implications

The results obtained in this study demonstrate that higher opioid consumption was associated with higher rate of PONV in nursing mothers as well as a higher incidence of neonatal adverse effects. Incorporating pharmacogenetic profiling into routine obstetric care can potentially further identify mothers at heightened risk for PONV and neonatal adverse effects; larger studies with higher numbers of CYP2D6 ultra-rapid and poor metabolizers are needed. Healthcare providers can personalize analgesic strategies to optimize safety and efficacy. Furthermore, reducing adverse effects, decreasing hospital length of stay, and lowering the incidence of opioid use can contribute to cost savings across multiple levels of healthcare. The findings further emphasize the value of multimodal analgesia within ERAS protocols, with opioids used only on an as-needed basis to lower opioid consumption without compromising pain control.

As seen in our results, widespread genotyping of parturient prior to delivery has potentially important implications for both maternal and neonatal outcomes. The clinical feasibility is promising in this population, given their proximity to healthcare and frequency of antenatal appointments, allowing for opportunities for testing and follow-up prior to delivery. The known concern surrounding perioperative pain control in cesarean deliveries may also make this population especially motivated to complete the genetic testing and follow up involved [28].

However, there are many practical limitations, including patient education and genetic counseling, regarding the results, third-party payer coverage, gestational time constraints, and the availability of results to providers at delivery. The involvement of genetic counselors would be helpful in educating patients both before and after testing about the utility and importance of personalized care. Due to evidence of systemic cost-savings, some third-party insurers have been providing coverage for pediatric perioperative CYP2D6 genotyping for prescription of oral opioids. The possibility to extend coverage to other populations may exist as more data continues to show a reduction in adverse events and, therefore, healthcare costs with genotyping prior to treatment across many drug classes [29]. Furthermore, without the ability to obtain genotypic information rapidly, patients undergoing emergency cesarean deliveries would be excluded from the implementation of personalized opioid prescription strategies in the immediate postoperative period.

### 4.5. Future Directions

Future research should focus on validating these findings in diverse populations at different large medical centers. The development of clinical decision-support tools that integrate pharmacogenetic data could further enhance the precision of pain management strategies, while more cost-reduction analyses could benefit the field by providing coverage incentives for third-party payers. Additionally, exploring potential epigenetic changes in opioid receptors or pain signaling in pregnancy and postpartum period would provide interesting context to opioid use in this population. Although some data suggests that higher neonatal opioid exposure is associated with poor neurodevelopmental outcomes, these studies focused on intravenous opioid use in extremely low-birth-weight infants and those undergoing pediatric cardiac surgery [30,31]. Longitudinal studies examining the long-term impact of neonatal opioid exposure at lower enteral doses are needed to inform evidence-based guidelines for safe opioid use during breastfeeding.

## 5. Conclusions

In this prospective clinical cohort study conducted under an opioid-sparing ERAS protocol for post-cesarean delivery pain management, no cases of respiratory depression were observed. Use of the lowest effective doses of opioids as needed within an opioid-sparing ERAS protocol may reduce the risk of opioid-related side effects in neonates. Our hypothesis-generating association between maternal CYP2D6 ultra-rapid metabolizer and risk of neonatal side effects warrants further investigation in larger cohorts. Preoperative genotyping may potentially help identify mothers and breastfed neonates at increased risk for opioid-related adverse outcomes if validated in a larger study with higher numbers of CYP2D6 poor and ultra-rapid metabolizers.

The use of multimodal analgesia within ERAS protocols provides an effective framework to reduce opioid consumption while maintaining adequate pain control. These findings advocate for further research to validate pharmacogenetic predictors in larger, multicenter cohorts and to explore non-opioid alternatives to enhance safety during breastfeeding. Longitudinal studies with robust pharmacokinetic measures are also essential to assess the developmental impact of higher neonatal opioid exposure. This hypothesis generating study contributes to the growing body of evidence supporting the need for large and robust precision medicine studies to improve maternal and neonatal outcomes and further advance the safety and efficacy of pain management in nursing mothers.

## Figures and Tables

**Table 1:** Baseline characteristics by postoperative nausea and vomiting (PONV) over postpartum days 0–3.

Variable	All n = 210	No PONV n = 183	PONV n = 27	*p*-Value
**Age (years)**, mean (SD)	32.1 (4.8)	32.1 (4.7)	31.9 (5.1)	**0.73**
**Race**				**0.025**
White	167 (80%)	150 (82%)	17 (63%)	
Black	26 (12%)	22 (12%)	4 (15%)	
Asian	10 (5%)	6 (3%)	4 (15%)	
Mixed/Other	7 (3%)	5 (3%)	2 (7%)	
**Ethnicity**—Hispanic or Latino	11 (5%)	8 (4%)	3 (11%)	**0.14**
**BMI**, mean (SD)	34.6 (7.3)	34.4 (7.4)	35.8 (7.0)	**0.20**
**Gravidity**	n = 192	n = 169	n = 23	**0.91**
Median (25th:75th)	2 (2:3)	2 (2:3)	2 (1:3)	
**Parity**	n = 193	n = 170	n = 23	**0.28**
Median (25th:75th)	1 (0:1)	1 (1:1)	1 (0:2)	
**Number of prior cesarean section**	n = 209	n = 182	n = 27	**0.10**
Median (25th:75th)	1 (0:1)	1 (0:1)	0 (0:1)	
**Any surgical history not related to cesarean section**	n = 208	n = 181	n = 27	**0.26**
Yes	118 (57%)	100 (55%)	18 (67%)	
**Was this a scheduled Cesarean Section?**	n = 202	n = 177	n = 25	**0.13**
Yes	167 (83%)	149 (84%)	18 (72%)	
**Anesthesia type**	n = 209	n = 183	n = 26	**0.87**
Spinal	178 (85%)	157 (86%)	21 (81%)	
CSE or epidural (de novo)	6 (3%)	5 (3%)	1 (4%)	
Epidural (pre-existing)	24 (11%)	20 (11%)	4 (15%)	
General (primary or converted to GA)	1 (0%)	1 (1%)	0 (0%)	
**Total Surgery Time,** mean (SD)	52.0 (15.5)	51.9 (15.7)	52.9 (14.5)	**0.77**
**Any Mental Illness**	118 (56%)	101 (55%)	17 (63%)	**0.45**
Anxiety or depression	116 (55%)	100 (55%)	16 (59%)	**0.65**
Bipolar disorder	5 (2%)	3 (2%)	2 (7%)	**0.07**
PTSD	14 (7%)	12 (7%)	2 (7%)	**0.87**
ADHD	8 (4%)	4 (2%)	4 (15%)	**0.001**
Other	6 (3%)	4 (2%)	2 (7%)	**0.13**
**Maternal CYP2D6 Phenotype**	n = 199	n = 175	n = 24	**0.31**
Normal or Intermediate	181 (91%)	161 (92%)	20 (83%)	
Ultra-rapid	7 (4%)	5 (3%)	2 (8%)	
Poor	11 (6%)	9 (5%)	2 (8%)	

**Table 2:** Maternal and neonatal clinical outcomes over postpartum days 0–3.

Variable	All n = 210
**Maternal Clinical Outcomes**	**n (%) or Mean (SD**)
Respiratory Depression	0 (0%)
Any Postoperative Nausea and Vomiting (PONV)	27 (13%)
Averaged Pain Score, mean (SD)	3.2 (1.6)
Max Pain Score, mean (SD)	4.9 (1.9)
In-hospital opioid use after delivery	161 (77%)
Total in-hospital opioid consumption in MME (mg), mean (SD)	44.4 (47.5)
Prolonged hospitalization (>3 days)	13 (6%)
**Neonatal Clinical Outcomes**	
Neonatal Composite Outcome of Opioid Side Effects (Respiratory depression monitoring, sedation, and limpness)	28 (13%)
Monitoring of Neonatal Respiratory Depression	8
Neonatal Sedation (Any nap > 4 h)	7
Neonatal Limpness (Any limpness > 0)	15
Neonatal Extended Length of Stay (>72 h)	77 (37%)
Neonates Exhibiting Opioid Withdrawal (Any SOWSS > 4)	3 (1%)

**Table 3:** Associations between total in-hospital MME and CYP2D6 phenotype on maternal PONV and neonatal composite outcome of opioid side effects (n = 210).

Predictors	Maternal PONV Outcome		
	n/N (%) PONV	Unadjusted Relative Risk (95%CI)	Adjusted * Relative Risk (95%CI)
**Total in-hospital MME**	**n = 210**	***p* = 0.49**	***p* = 0.36**
0 mg	4/49 (8.2%)	Reference	Reference
0–70 mg	15/112 (13.4%)	1.64 (0.57–4.70)	1.89 (0.61–5.85)
>70 mg	8/49 (16.3%)	2.00 (0.64–6.24)	2.56 (0.70–9.29)
**Maternal CYP2D6 Phenotype**	**n = 199**	***p* = 0.26**	***p* = 0.26**
Normal or Intermediate	20/181 (11.0%)	Reference	Reference
Poor	2/11 (18.2%)	1.65 (0.44–6.22)	1.86 (0.49–7.01)
Ultra-rapid	2/7 (28.6%)	2.62 (0.76–9.04)	3.78 (0.65–22.05)
**Predictors**	**Neonatal composite outcome of opioid side effects** **(RD monitoring, sedation, and limpness)**		
	**n/N (%) composite outcome**	**Unadjusted** **Relative Risk (95%CI)**	Adjusted * **Relative Risk (95%CI)**
**Total in-hospital MME**	**n = 210**	***p* = 0.07**	***p* = 0.07**
0 mg	3/49 (6.1%)	Reference	Reference
0–70 mg	14/112 (12.5%)	2.04 (0.61–6.84)	1.79 (0.52–6.22)
>70 mg	11/49 (22.4%)	3.67 (1.08–12.44)	3.79 (1.01–14.28)
**Maternal CYP2D6 Phenotype**	**n = 199**	***p* = 0.93**	***p* = 0.93**
Normal or Intermediate	24/181 (13.3%)	Reference	Reference
Poor	1/11 (9.1%)	0.69 (0.10–4.66)	0.71 (0.11–4.69)
Ultra-rapid	1/7 (14.3%)	1.07 (0.17–6.96)	1.14 (0.15–8.50)

* Adjusted models (n = 199) accounted for maternal characteristics including age, non-White race, BMI, any mental illness, and average pain score over three postpartum days.

**Table 4:** Associations between CYP2D6 phenotype and average pain scores with total in-hospital MME (n = 210).

Predictors	Total In-Hospital Opioid Use in MME (mg)		
	Mean (SD) of Total MME	Unadjusted Mean Ratio (95%CI)	Adjusted * Mean Ratio (95%CI)
**Average Pain Score**		***p* = 0.002**	***p* = 0.01**
0–3	31.3 (35.3)	Reference	Reference
4+	78.6 (57.3)	2.51 (1.42–4.43)	2.44 (1.29–4.61)
**Maternal CYP2D6 Phenotype**	**n = 199**	***p* = 0.96**	***p* = 0.92**
Normal or Intermediate	43.2 (45.8)	Reference	Reference
Poor	51.1 (72.1)	1.19 (0.36–3.89)	1.09 (0.34–3.50)
Ultra-rapid	42.9 (44.9)	1.03 (0.23–4.61)	0.75 (0.17–3.38)

* Adjusted models (n = 199) accounted for maternal characteristics including age, non-White race, BMI, and any mental illness.

## Data Availability

The raw data supporting the conclusions of this article will be made available by the authors on request.

## Appendix A

**Table A1:** Maternal and neonatal CYP2D6 metabolizer phenotypes.

	All n = 190	CYP2D6 Metabolizer Phenotype (Maternal)				
		Normal n = 105 (55.3%)	Ultra-Rapid n = 6 (3.2%)	Intermediate n = 63 (33.2%)	Poor n = 11 (5.8%)	Unknown n = 5 (2.6%)
**CYP2D6 Metabolizer Phenotype** **(Infant)**	n = 190	n = 105	n = 6	n = 63	n = 11	n = 5
Normal	104 (54.7%)	74 (70.5%)	5 (83.3%)	22 (34.9%)	0 (0.0%)	3 (60.0%)
Ultra-rapid	5 (2.6%)	3 (2.9%)	1 (16.7%)	1 (1.6%)	0 (0.0%)	0 (0.0%)
Intermediate	66 (34.7%)	24 (22.9%)	0 (0.0%)	31 (49.2%)	10 (90.9%)	1 (20.0%)
Poor	7 (3.7%)	0 (0.0%)	0 (0.0%)	6 (9.5%)	1 (9.1%)	0 (0.0%)
Unknown	8 (4.2%)	4 (3.8%)	0 (0.0%)	3 (4.8%)	0 (0.0%)	1 (20.0%)

**Table A2:** Maternal clinical outcomes over time.

Variable	PPD0/PACU n = 210	PPD1 n = 210	PPD2 n = 210	PPD3 n = 210	Discharge n = 210
**Respiratory Depression**	n = 209	n = 210	n = 202	n = 119	
Yes	0 (0%)	0 (0%)	0 (0%)	0 (0%)	
**PONV**	n = 210	n = 210	n = 210	n = 210	
Yes	23 (11%)	5 (2%)	0 (0%)	0 (0%)	
**Did the patient take opioids**	n = 210	n = 210	n = 209	n = 206	
No	121 (58%)	82 (39%)	76 (36%)	147 (71%)	
Yes	89 (42%)	128 (61%)	133 (64%)	59 (29%)	
**In-Hospital Opioid MME (mg) ***	n = 161	n = 161	n = 161	n = 161	
Mean (SD)	8.0 (9.8)	20.7 (19.2)	21.9 (19.3)	7.3 (13.0)	
Median (25th:75th)	7.5 (0.0:15.0)	15.0 (7.5:30.0)	15.0 (7.5:30.0)	0.0 (0.0:7.5)	
Min:Max	0.0:45.0	0.0:90.0	0.0:90.0	0.0:75.0	
**NRS Pain**	n = 209	n = 210	n = 209	n = 207	n = 204
Mean (SD)	1.7 (2.3)	3.8 (2.2)	4.0 (2.0)	3.3 (1.8)	3.5 (1.9)
Median (25th:75th)	0.0 (0.0:3.0)	4.0 (2.0:5.0)	4.0 (3.0:5.0)	3.0 (2.0:5.0)	3.0 (2.0:5.0)
Min:Max	0.0:10.0	0.0:10.0	0.0:10.0	0.0:8.0	0.0:9.0
**NRS Pain**	n = 209	n = 210	n = 209	n = 207	n = 204
0	109 (52%)	18 (9%)	11 (5%)	11 (5%)	11 (5%)
1	20 (10%)	12 (6%)	2 (1%)	22 (11%)	16 (8%)
2	22 (11%)	26 (12%)	36 (17%)	38 (18%)	33 (16%)
3	14 (7%)	44 (21%)	45 (22%)	42 (20%)	50 (25%)
4	12 (6%)	38 (18%)	33 (16%)	42 (20%)	37 (18%)
5	15 (7%)	30 (14%)	38 (18%)	23 (11%)	31 (15%)
6	6 (3%)	16 (8%)	24 (11%)	22 (11%)	15 (7%)
7	5 (2%)	12 (6%)	9 (4%)	4 (2%)	3 (1%)
8	4 (2%)	11 (5%)	6 (3%)	3 (1%)	6 (3%)
9	0 (0%)	1 (0%)	4 (2%)	0 (0%)	2 (1%)
10	2 (1%)	2 (1%)	1 (0%)	0 (0%)	0 (0%)

* In-hospital opioid MME was reported among 161 mothers who had taken any opioids over PPD0–3.

## References

[1] Cahill A.G., Raghuraman N., Gandhi M., Kaimal A.J. (2024). First and Second Stage Labor Management: ACOG Clinical Practice Guideline No. 8. Obstet. Gynecol..

[2] Antoine C., Young B.K. (2020). Cesarean section one hundred years 1920–2020: The Good, the Bad and the Ugly. J. Perinat. Med..

[3] Barber E.L., Lundsberg L.S., Belanger K., Pettker C.M., Funai E.F., Illuzzi J.L. (2011). Indications contributing to the increasing cesarean delivery rate. Obstet. Gynecol..

[4] Peahl A.F., Smith R., Johnson T.R.B., Morgan D.M., Pearlman M.D. (2019). Better late than never: Why obstetricians must implement enhanced recovery after cesarean. Am. J. Obstet. Gynecol..

[5] Lim G., Facco F.L., Nathan N., Waters J.H., Wong C.A., Eltzschig H.K. (2018). A Review of the Impact of Obstetric Anesthesia on Maternal and Neonatal Outcomes. Anesthesiology.

[6] Horn R., Hendrix J.M., Kramer J. (2025). Postoperative Pain Control.

[7] Crews K.R., Gaedigk A., Dunnenberger H.M., Leeder J.S., Klein T.E., Caudle K.E., Haidar C., Shen D.D., Callaghan J.T., Sadhasivam S. (2014). Clinical Pharmacogenetics Implementation Consortium guidelines for cytochrome P450 2D6 genotype and codeine therapy: 2014 update. Clin. Pharmacol. Ther..

[8] Silverman M., Zwolinski N., Wang E., Lockwood N., Ancuta M., Jin E., Li J. (2023). Regional Analgesia for Cesarean Delivery: A Narrative Review Toward Enhancing Outcomes in Parturients. J. Pain Res..

[9] Bryant A.S., Miller R.S. (2021). Pharmacologic Stepwise Multimodal Approach for Postpartum Pain Management: ACOG Clinical Consensus No. 1. Obstet. Gynecol..

[10] Pharmacologic Stepwise Multimodal Approach for Postpartum Pain Management. https://www.acog.org/clinical/clinical-guidance/clinical-consensus/articles/2021/09/pharmacologic-stepwise-multimodal-approach-for-postpartum-pain-management.

[11] Pesonen A., Hakomäki H., Kokki H., Ranta V.P., Rinne V., Kokki M. (2024). Breast milk oxycodone concentrations in mothers given oxycodone for post-Caesarean delivery pain management. Br. J. Clin. Pharmacol..

[12] Lam J., Kelly L., Ciszkowski C., Landsmeer M.L., Nauta M., Carleton B.C., Hayden M.R., Madadi P., Koren G. (2012). Central nervous system depression of neonates breastfed by mothers receiving oxycodone for postpartum analgesia. J. Pediatr..

[13] Baber M., Bapat P., Nichol G., Koren G. (2016). The pharmacogenetics of opioid therapy in the management of postpartum pain: A systematic review. Pharmacogenomics.

[14] Smolina K., Weymann D., Morgan S., Ross C., Carleton B. (2015). Association between regulatory advisories and codeine prescribing to postpartum women. JAMA.

[15] Gaedigk A., Sangkuhl K., Whirl-Carrillo M., Klein T., Leeder J.S. (2017). Prediction of CYP2D6 phenotype from genotype across world populations. Genet. Med..

[16] Badreldin N., Grobman W.A., Chang K.T., Yee L.M. (2018). Opioid prescribing patterns among postpartum women. Am. J. Obstet. Gynecol..

[17] Kallen A.N. (2018). ACOG Committee Opinion No. 750: Perioperative Pathways: Enhanced Recovery After Surgery. Obstet Gynecol..

[18] Brat G.A., Agniel D., Beam A., Yorkgitis B., Bicket M., Homer M., Fox K.P., Knecht D.B., McMahill-Walraven C.N., Palmer N. (2018). Postsurgical prescriptions for opioid naive patients and association with overdose and misuse: Retrospective cohort study. BMJ.

[19] Levy H.A., Karamian B.A., Canseco J.A., Henstenburg J., Larwa J., Haislup B., Kaye I.D., Woods B.I., Radcliff K.E., Hilibrand A.S. (2023). Does a High Postoperative Opioid Dose Predict Chronic Use After ACDF?. World Neurosurg..

[20] Stevens J.C., Marsh S.A., Zaya M.J., Regina K.J., Divakaran K., Le M., Hines R.N. (2008). Developmental changes in human liver CYP2D6 expression. Drug Metab. Dispos..

[21] Ruggiero A., Ariano A., Triarico S., Capozza M.A., Ferrara P., Attinà G. (2019). Neonatal pharmacology and clinical implications. Drugs Context.

[22] Hines R.N. (2007). Ontogeny of human hepatic cytochromes P450. J. Biochem. Mol. Toxicol..

[23] Pokela M.L., Olkkola K.T., Seppälä T., Koivisto M. (1993). Age-related morphine kinetics in infants. Dev. Pharmacol. Ther..

[24] Lynn A.M., Slattery J.T. (1987). Morphine pharmacokinetics in early infancy. Anesthesiology.

[25] Bhat R., Chari G., Gulati A., Aldana O., Velamati R., Bhargava H. (1990). Pharmacokinetics of a single dose of morphine in preterm infants during the first week of life. J. Pediatr..

[26] Lacroix D., Sonnier M., Moncion A., Cheron G., Cresteil T. (1997). Expression of CYP3A in the human liver—Evidence that the shift between CYP3A7 and CYP3A4 occurs immediately after birth. Eur. J. Biochem..

[27] Romberg R.R., Olofsen E., Bijl H., Taschner P.E., Teppema L.J., Sarton E.Y., van Kleef J.W., Dahan A. (2005). Polymorphism of mu-opioid receptor gene (OPRM1:c.118A>G) does not protect against opioid-induced respiratory depression despite reduced analgesic response. Anesthesiology.

[28] Carvalho B., Cohen S.E., Lipman S.S., Fuller A., Mathusamy A.D., Macario A. (2005). Patient preferences for anesthesia outcomes associated with cesarean delivery. Anesth. Analg..

[29] Plumpton C.O., Roberts D., Pirmohamed M., Hughes D.A. (2016). A Systematic Review of Economic Evaluations of Pharmacogenetic Testing for Prevention of Adverse Drug Reactions. Pharmacoeconomics.

[30] O’Byrne M.L., Baxelbaum K., Tam V., Griffis H., Pennington M.L., Hagerty A., Naim M.Y., Nicolson S.C., Shillingford A.J., Sutherland T.N. (2024). Association of Postnatal Opioid Exposure and 2-Year Neurodevelopmental Outcomes in Infants Undergoing Cardiac Surgery. J. Am. Coll. Cardiol..

[31] Kocek M., Wilcox R., Crank C., Patra K. (2016). Evaluation of the relationship between opioid exposure in extremely low birth weight infants in the neonatal intensive care unit and neurodevelopmental outcome at 2 years. Early Hum. Dev..

